# Supplementary material for: Computer-aided drug design to generate a unique antibiotic family
Source: Nat Commun. 2024 Sep 27;15:8317. doi: 10.1038/s41467-024-52797-2 (PMC11436758; doi:10.1038/s41467-024-52797-2)
Supplement: Supplementary file 1 — Supplementary Information [file 41467_2024_52797_MOESM1_ESM.pdf]

**Supplementary Information**

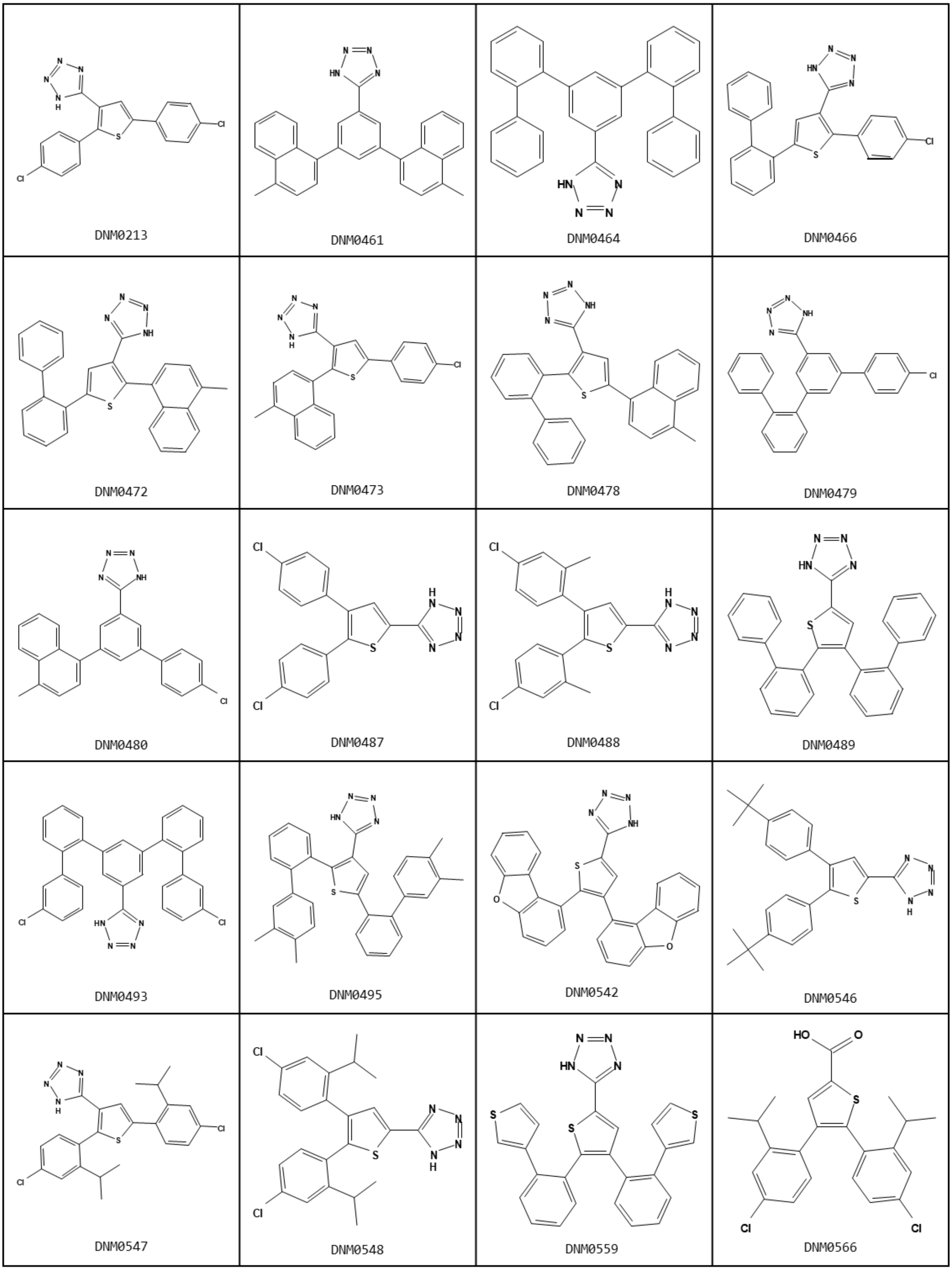


**Supplementary Figure 1. Structures of the DNM compounds used in this study.**

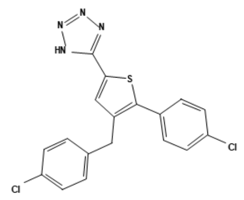

DNM0579

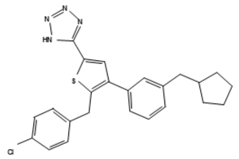

DNM0610

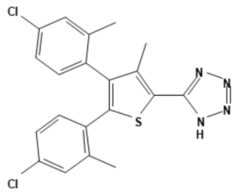

DNM0614

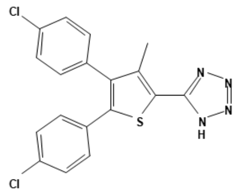

DNM0619

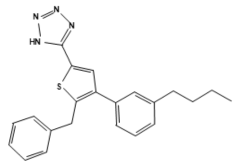

DNM0626

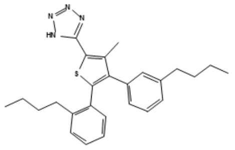

DNM0627

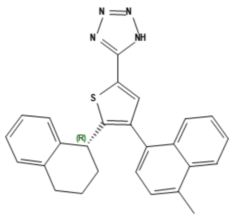

DNM0636

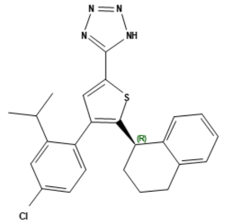

DNM0637

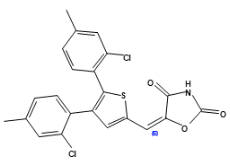

DNM0644

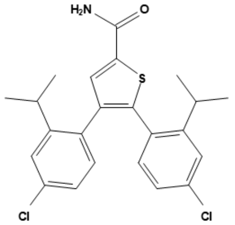

DNM0650

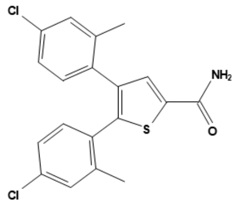

DNM0652

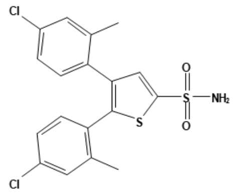

DNM0658

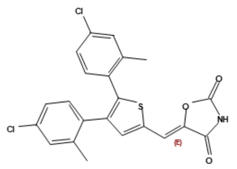

DNM0663

**Supplementary Figure 1 (continued)**

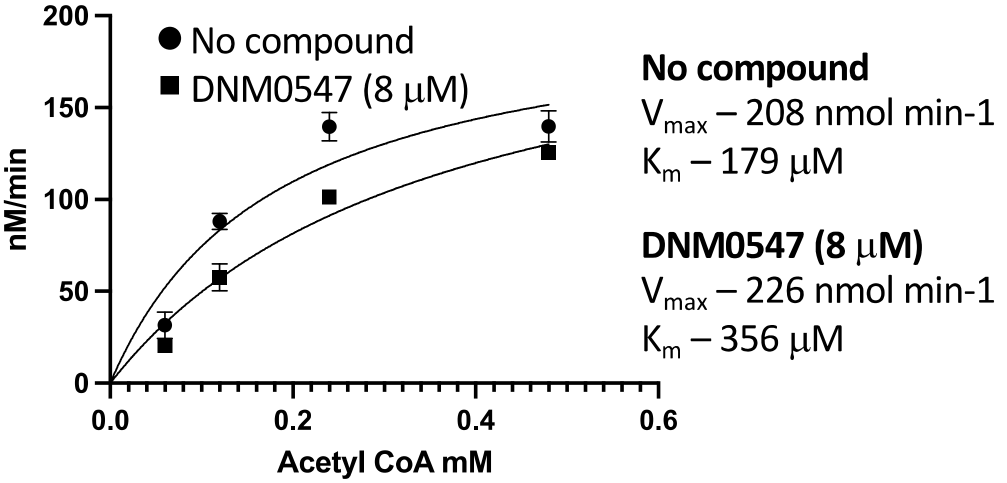


**Supplementary Figure 2. DNM0547 competitively inhibits AcpS..** AcpS enzyme activity was determined versus using purified AcpS and increasing concentrations of CoA in the presence and absence of 8 μM DNM0547**.** Data are the mean values +/- SD of three separate experiments performed in triplicate from three separate enzyme preparation. GraphPad Prism was used to determine $V_{max}$ and $K_m$ values.

**a**

| AcpS variant | Ac3vity (nmol min$^{-1}$ mg$^{-1}$) | % Wild Type Ac3vity |
|---|---|---|
| AcpS wt | 550 +/- 25 | 100 |
| AcpS$^{R48A}$ | 1281 +/- 19 | 232 |
| AcpS$^{F50A}$ | ND* | 0 |
| AcpS$^{R53A}$ | ND | 0 |
| AcpS$^{K62A}$ | ND | 0 |
| AcpS$^{N81A}$ | 101 +/- 9 | 18 |
| AcpS$^{H108A}$ | 412 +/- 12 | 75 |

*ND, not detectable

**b**

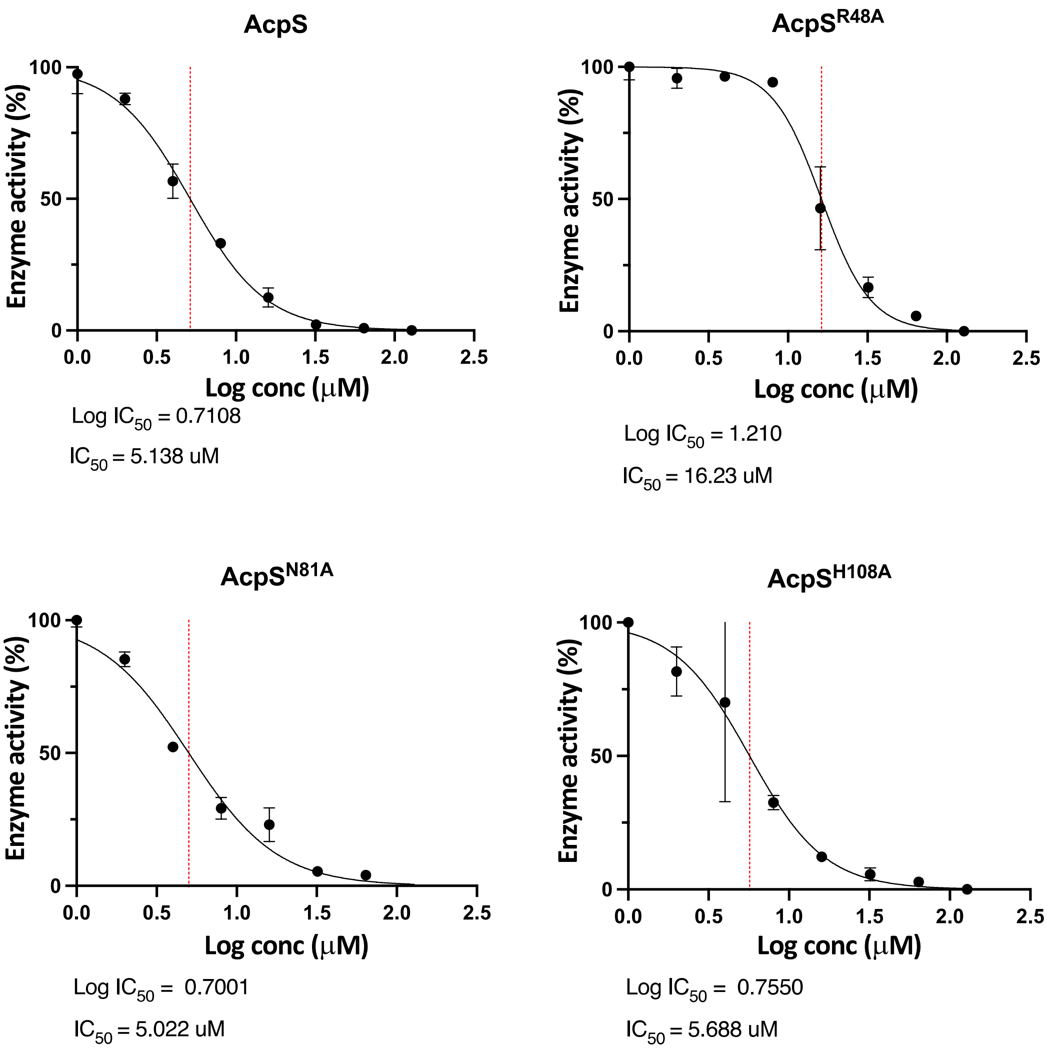


**Supplementary Figure 3. Inhibition of AcpS and site-directed mutants by DNM0547. a,** Activity of *E. coli* wild type and AcpS mutants determined using purified proteins. **b,** AcpS enzyme activity for each AcpS mutant was assayed at increasing concentration of DNM0547 (0-128 μM). $IC_{50}$ was determined using GraphPad Prism. The data for each variant is represented relative to its non-inhibited condition. *E. coli* amino acid numbering is displayed*;* *E. coli* compared to analogous *S. aureus* amino acids residues are R49, R46; F50, F50; R53, R54; K62, K63; N81, N82; H108, N/A, respectively. All experiments were performed three times in triplicate and expressed and mean +/- SD using students t-test.

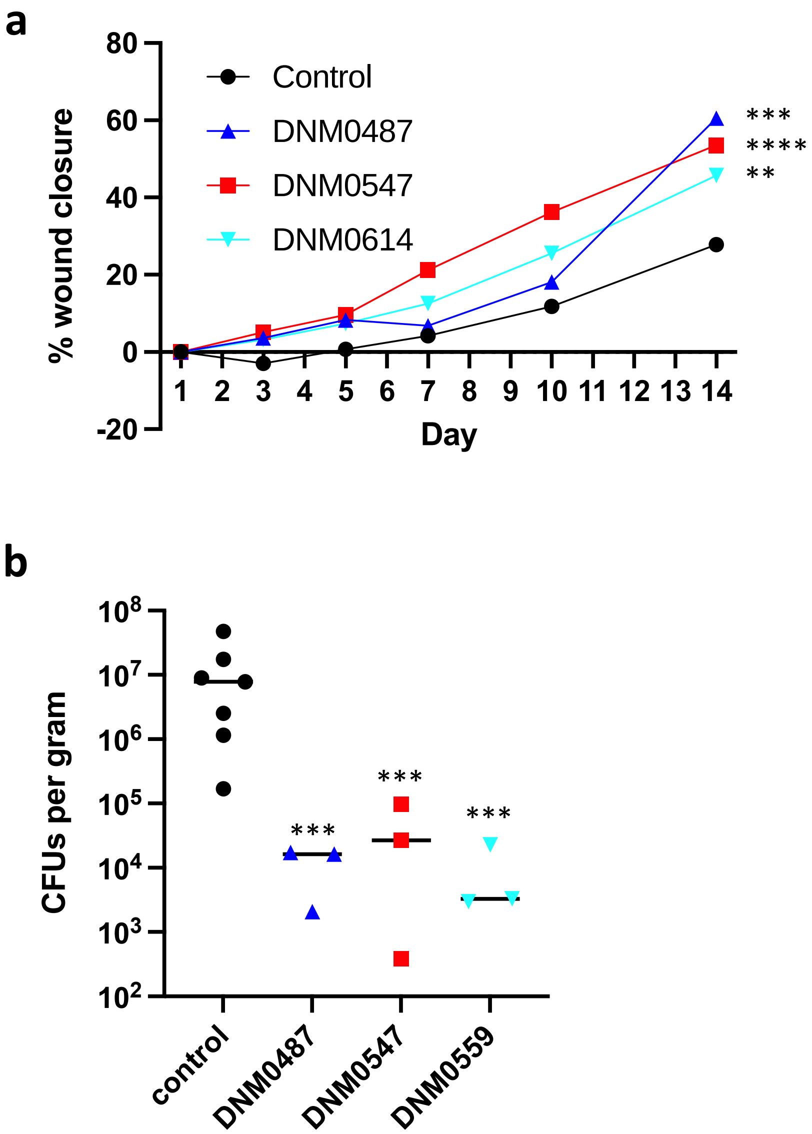


**Supplementary Figure 4.** ***In vivo*** **efficacy of DNM compounds. a**, Activity of DNM compounds against MRSA (ATCC 33591) in a rabbit ischemic ear model of infection; control wounds (n=11), DNM0487 (n=3), DNM0547 (n=10), DNM0614 (n=3). Data are represented as means with significance determined at day 14 using an unpaired student's t-test with Welch's correction,***$P<0.05$, ****$P<0.04$, *****$P<0.03$. **b**, Colony forming units (CFUs) per gram of tissue were determined at day 14 of the rabbit ischemic ear model of infection. Data are represented as individual data points with the line representing the mean. Significance was determine using an unpaired one-tailed student's t-test with Welch's correction, *** $P<0.007$.

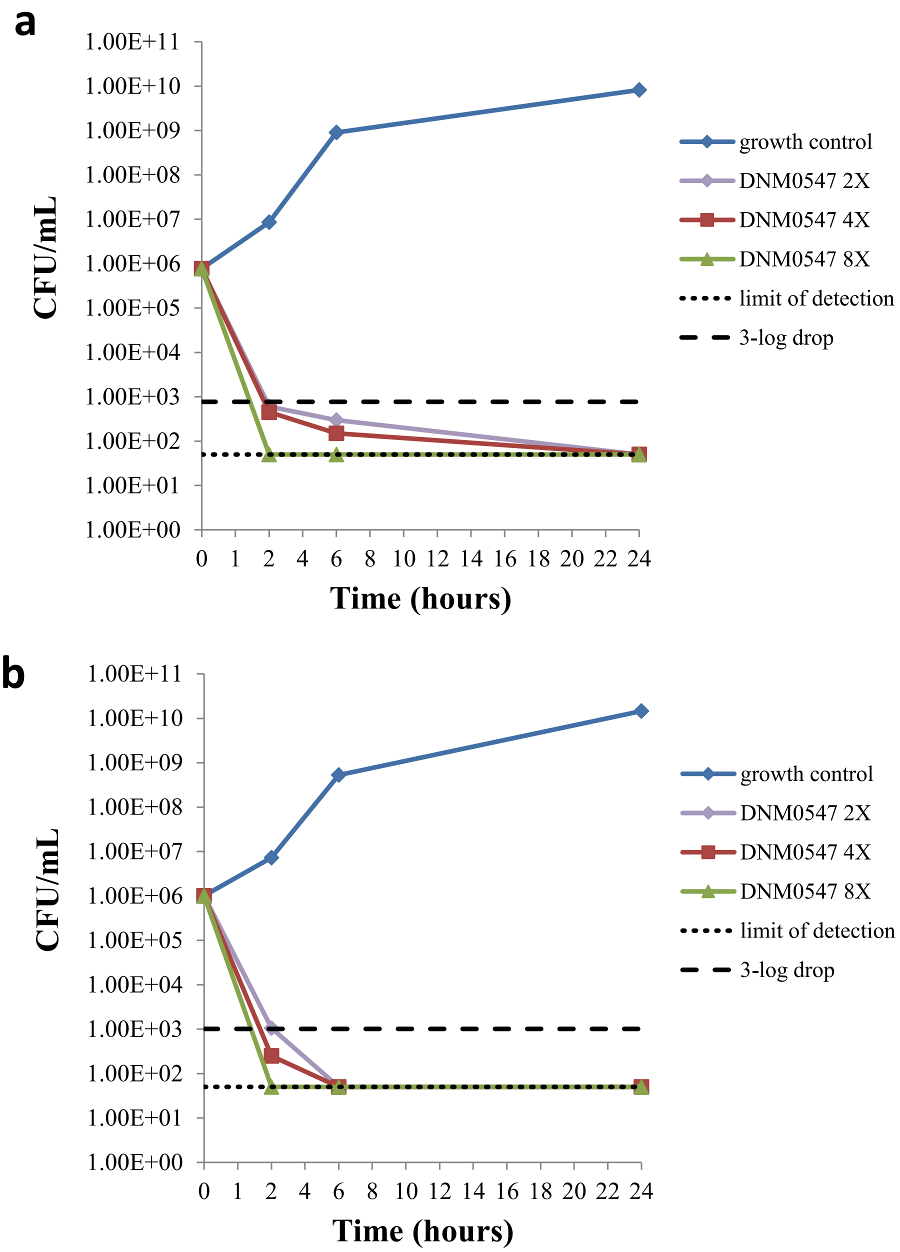


**Supplementary Figure 5. Time kill of DNM0547 versus *S. aureus*.** The time kill of DNM0547 at 2X, 4X, and 8X the MIC against **a**, MRSA MMX 2119 (NRS23, USA100) and **b**, MRSA MMX 4675 (ATCC BAA-1556, USA300) were determined. Representative experiments are shown.

**a**

| Compound | MBP | MBP-AcpS |
|---|---|---|
| DNM0547 | 32 | 256 |

**b** **c**

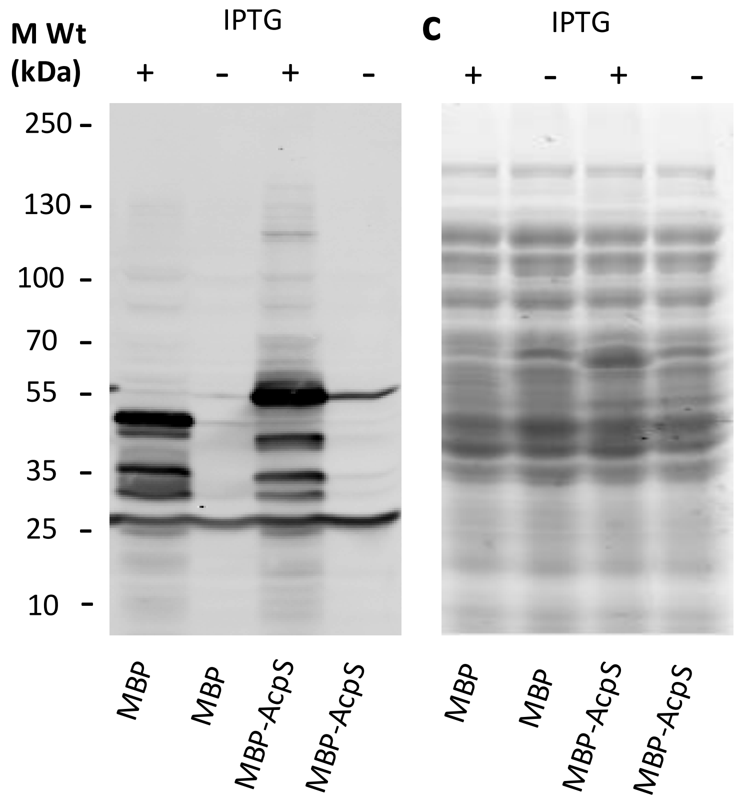


**Supplementary Figure 6. Increased expression of AcpS increases the MIC of DNM0547. a**, the minimum inhibitory concentration (MIC; µg/ml) of the D22 strain of *E. coli* upon expression of plasmid borne maltose binding protein (MBP) AcpS from an IPTG inducible promoter versus just over-expression of MBP only. The D22 strain of *E. coli* has a porous outer membrane due to a point mutation in the lpxC gene enabling some compounds to enter the cell that can not permeate wild type *E. coli*. AcpS is expressed as a fusion with MBP. MICs were determined upon IPTG induction of MBP-AcpS with expression of just MBP used as control. **b**, western blot against MBP (New England Biolabs E8032S, 1:1000 dilution) to assess expression; IPTG induced expression of MBP and MBP-AcpS at the expected M Wts. **c**, equal protein was added from each *E. coli* protein extract used for the western blot. Ponceau stain of the membrane to assess protein loading and transfer.

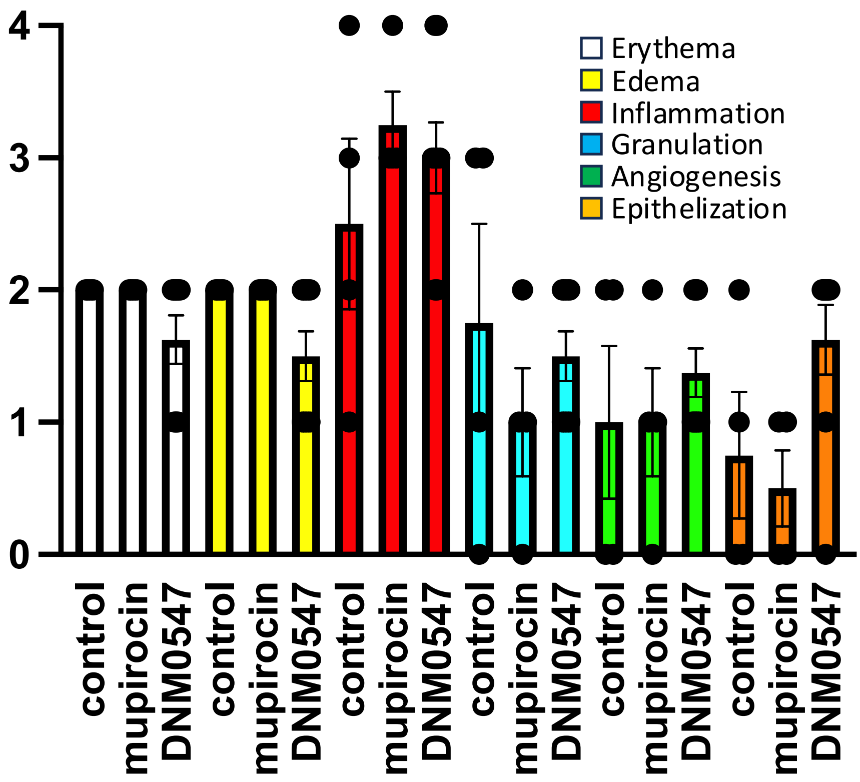


**Supplementary Figure 7. Pathology of rabbit ischemic ear wounds after treatment with DNM0547.** The wound was placed into a container with 10 % neutral buffered formalin and processed for routine H&E histological evaluation. Wounds were scored on a scale of 0-4 at the wound site for erythema, edema, inflammation, granulation, angiogenesis, and epithelialization in a blinded manner by a board certified member of the College of American Pathologists. A minimum of four animals/wounds per treatment were analyzed with individual values displayed. Mean +/- SEM are shown.

**a**

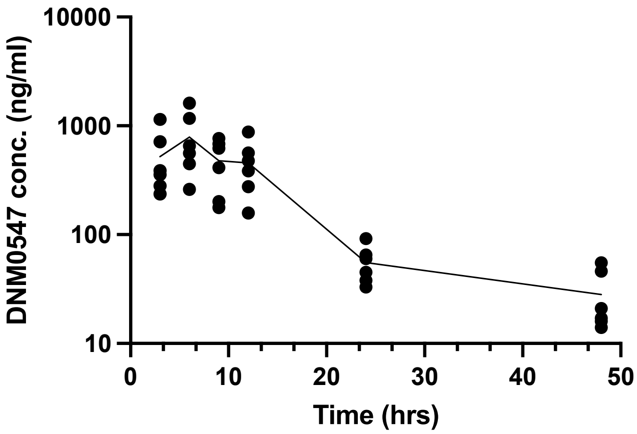


**b**

| Parameter | Value (mean) | CV (%) |
|---|---|---|
| **$Time_{1/2}$ (hrs)** | 8.78 | 19% |
| **$Time_{max}$ (hrs)** | 7.00 | 44% |
| **$Conc_{max}$ (ng/ml)** | 1020 | 37% |

**Supplementary Figure 8. Dermal PK of DNM0547. a**, 2% DNM0547 was applied to shaved skin of Sprague Dawley rats and DNM0547 concentration in serum was determined over time (n=5). **b**, PK parameters for DNM0547 in serum. CV, coefficient variation was determined using Graphpad Prism. Individual values for each experiments are displayed.

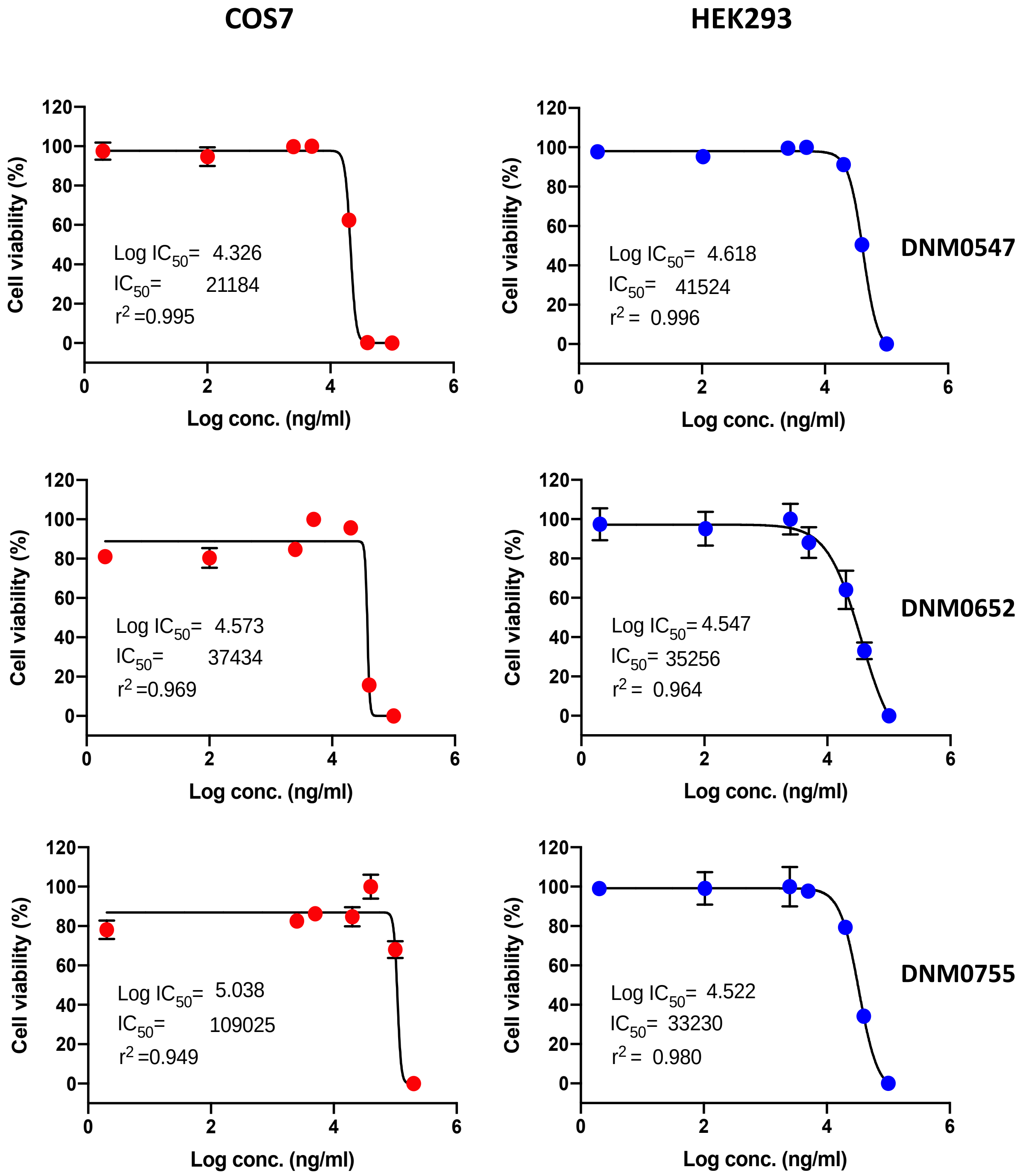


**Supplementary Figure 9. Toxicity of DNM compounds to cells in culture.** COS7 and HEK293 cells were grown in the presence of DNM compounds at the indicated concentrations for 24 hrs after which AlamarBlue reagent was added; cells were incubated a further 3.5 hrs and absorbance was determined at 580 nm and 600 nm to assess cell viability. Data are the mean values +/- SD of three separate experiments performed in triplicate.

**Chemical synthesis**

DNM0547 is 5-(2,5-bis(4-chloro-2-isopropylphenyl)thiophen-3-yl)-1H-tetrazole:

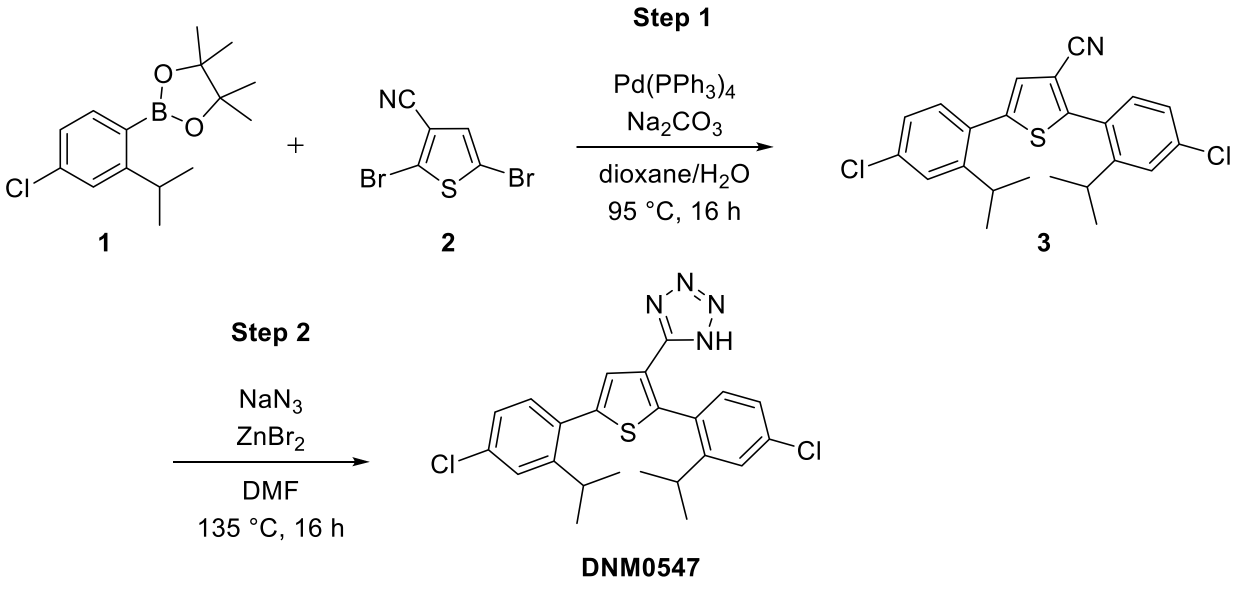


**Step 1:** To a stirred solution of 2-bromo-3-carbonitril-5-bromothiophene **(2**, 1.0 g, 3.7 mmol) in dioxane (20 mL), were added 4-chloro-2-isopropylphenylboronic ester **(1**, 2.31 g, 8.2 mmol, 2.2 eq), and 2M aq. sodium carbonate solution (9.4 mL, 18.8 mmol, 5 eq) at RT. The reaction mixture was degassed for 5 min with nitrogen before the addition of $Pd(PPh_3)_4$ (0.22 g, 0.19 mmol, 0.05 eq) and degassing continued for another 5 min. The reaction mixture was stirred at 95°C for 16 h. The dark reaction mixture was passed through celite bed and the bed was further washed with EtOAc (50 mL). The filtrate was washed with water (30 mL) and brine (30 mL). The organic layer was dried over anhydrous $Na_2SO_4$, filtered and the filtrate was concentrated under reduced pressure. The crude product was purified using flash column chromatography (25% DCM in hexanes) to obtain coupled product **3**.Yield: 1.4 g (90%)

**Step 2:** To a stirred solution of **3** (1.0 g, 2.4 mmol) in anhydrous DMF (20 mL), were added sodium azide (0.39 g, 6.0 mmol, 2.5 eq), and zinc bromide (1.36 g, 6.0 mmol, 2.5 eq) under nitrogen atmosphere. The reaction mixture was stirred at 135°C for 16 h. The reaction mixture was quenched with 1 N HCl solution (5 mL) and extracted with EtOAc (30 mL x 2). The combined organic layers were washed with brine (30 mL) and dried over anhydrous $Na_2SO_4$, filtered and the filtrate was concentrated under reduced pressure The crude product was purified using flash column chromatography (1% AcOH and 35% EtOAc in hexanes) to afford **DNM0547**. Yield: 0.88 g (80%). $^1$H NMR (500 MHz, $CDCl_3$): δ (ppm) 1.13 (s, br, 6H), 1.30 (d, *J* = 6.8 Hz, 6H), 2.92 (m, 1H), 3.38 (m, 1H), 7.26 (dd, *J* = 2.0, 8.1 Hz, 1H), 7.40 (m, 4H), 7.54 (d, *J* = 1.3 Hz, 1H), 7.76 (s, 1H); $^{13}$C NMR (125 MHz, $CDCl_3$): δ (ppm) 24.6 (2), 24.7, 24.8, 30.4, 31.0, 123.0. 126.5, 126.9, 127.2, 127.8. 128.1, 128.7, 130.0, 132.5, 132.7, 135.9, 137.8, 142.5, 143.9, 150. 151.5. (**Suppl Fig 10**).

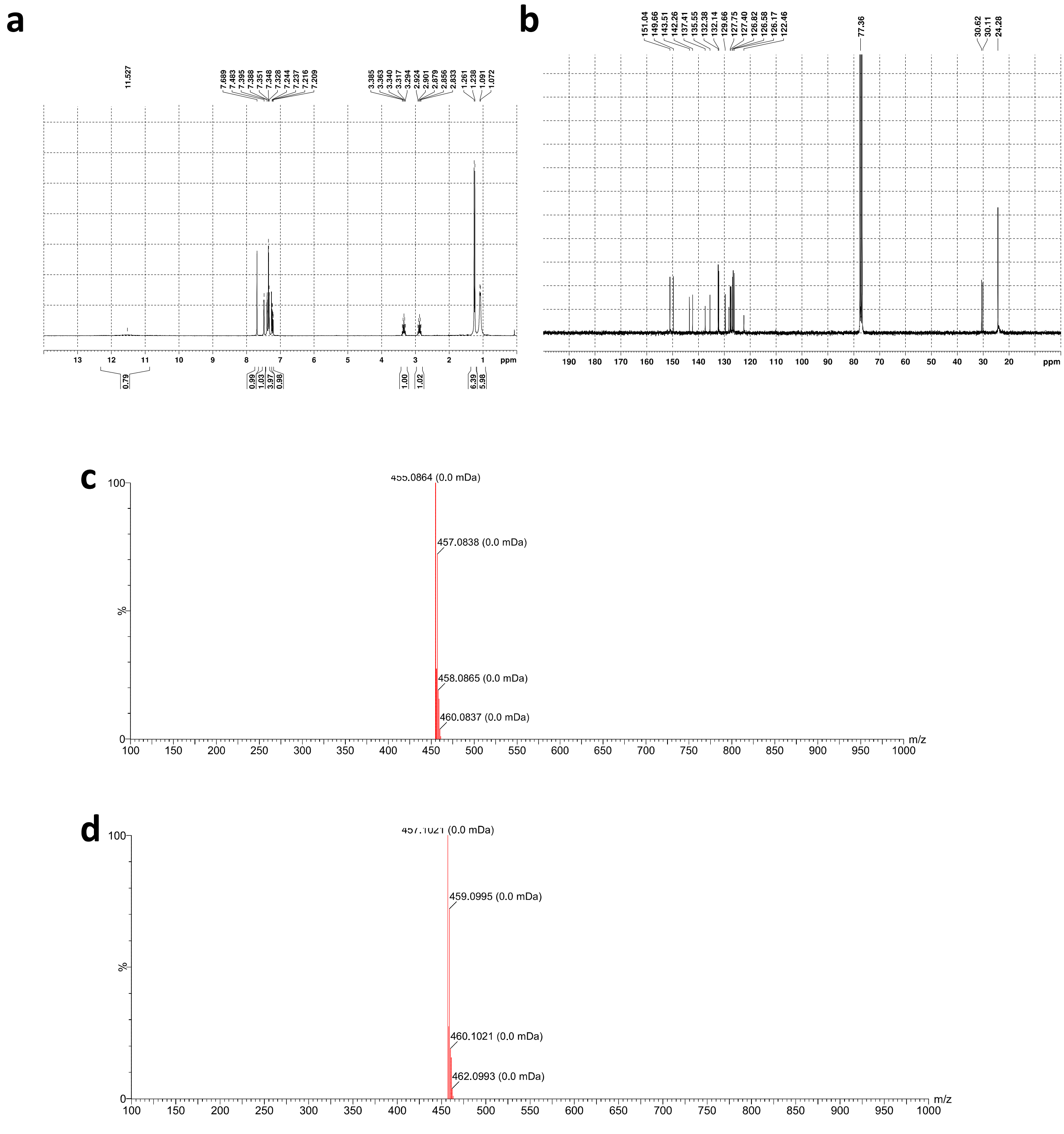


**Supplementary Figure 10. Nuclear magnetic spectra and mass spectrometry of DNM0547. a**, 1H spectra ere recorded on a Bruker Ultrashield at 300 MHz using residual non-deuterated solvent as an internal reference Chemical shifts are reported in parts per million (ppm) from tetramethysilane with the residual protic solvent resonance as internal standard. **b**, 13C NMR spectra were recorded on a Bruker Ultrashield at 75 MHz with complete proton decoupling. **c, d,** Tandem mass spectrometric data for DNM0547, collected using a liquid chromatograph coupled to a Waters Xevo G2-XS quadrupole time-of-flight mass spectrometer in negative and positive electrospray ionization modes, respectively. Most abundant isotopic species: $C_{23}H_{22}Cl_2N_4S$ negative predicted 455.0862, found 455.0862; $C_{23}H_{22}Cl_2N_4S$ positive predicted 457.1022, found 457.1022.

**Supplementary Table 1. Minimum inhibitory concentrations (MIC; μg/ml) for DNM compounds versus MRSA (ATCC 33591) and AcpS enzyme activity inhibitory concentration $IC_{50}$ (μM). MIC and IC50 values were determined from three individual replicates.**

| Compound | MIC | AcpS IC50 |
|---|---|---|
| | | |
| DNM0213 | 2 | 9 |
| DNM0461 | 0.5 | 7 |
| DNM0464 | 0.5 | 9 |
| DNM0466 | 1 | 9 |
| DNM0472 | 2 | 10 |
| DNM0473 | 2 | 10 |
| DNM0478 | 1 | 8 |
| DNM0479 | 1 | 9 |
| DNM0480 | 2 | 8 |
| DNM0487 | 2 | 1 |
| DNM0488 | 2 | 9 |
| DNM0489 | 2 | 10 |
| DNM0493 | 2 | 14 |
| DNM0495 | 2 | 10 |
| DNM0542 | 1 | 12 |
| DNM0546 | 2 | 16 |
| DNM0547 | 2 | 8 |
| DNM0548 | 2 | 8 |
| DNM0559 | 1 | 9 |
| DNM0566 | 1 | 13 |
| DNM0579 | 1 | 14 |
| DNM0610 | 1 | 9 |
| DNM0614 | 1 | 13 |
| DNM0619 | 1 | 14 |
| DNM0626 | 0.5 | 9 |
| DNM0627 | 0.25 | 10 |
| DNM0636 | 2 | 13 |
| DNM0637 | 2 | 10 |
| DNM0644 | 1 | 13 |
| DNM0663 | 1 | 15 |
| DNM0752 | 2 | 10 |
| DNM0753 | 2 | 1 |
| DNM0755 | 2 | 2 |
| DNM0759 | 2 | 5 |

**Supplementary Table 2. Minimum inhibitory concentrations (MIC; μg/ml) for DNM0547 and current antibiotics versus clinical and laboratory bacterial strains.**

| Organism (Phenotype) | Culture Number/Strain | DNM0547 | vancomycin | mupirocin | ciprofloxacin | erythromycin |
|---|---|---|---|---|---|---|
| *S. aureus* (MRSA) | 2167 | 1 | 0.5 | 0.12 | >16 | >16 |
| *S. aureus* (MRSA) | 2168 | 1 | 0.5 | 0.25 | >16 | >16 |
| *S. aureus* (MRSA) | 2169 | 2 | 1 | 0.25 | >16 | >16 |
| *S. aureus* (MRSA) | 2170 | 1 | 0.5 | 0.25 | >16 | >16 |
| *S. aureus* (MRSA) | 2171 | 1 | 0.5 | 0.25 | >16 | >16 |
| *S. aureus* (MRSA) | 2293 | 2 | 0.5 | 0.25 | 1 | >16 |
| *S. aureus* (MRSA) | 2294 | 1 | 0.5 | 0.25 | >16 | >16 |
| *S. aureus* (MRSA) | 2295 | 1 | 0.5 | 0.25 | 0.5 | >16 |
| *S. aureus* (MRSA) | 2296 | 1 | 0.5 | 0.25 | >16 | 0.5 |
| *S. aureus* (MRSA) | 2297 | 2 | 1 | 0.25 | 0.5 | >16 |
| *S. aureus* (MRSA) | NRS384/2011 | 2 | 0.5 | 1 | 4 | >16 |
| *S. aureus* (MRSA) | NRS382/2119 | 1 | 1 | 1 | >16 | >16 |
| *S. aureus* (MRSA) | NRS383/2120 | 1 | 0.5 | >16 | >16 | >16 |
| *S. aureus* (MRSA) | 3880 | 1 | 0.5 | >16 | 16 | >16 |
| *S. aureus* (MRSA) | 5373 | 1 | 0.5 | 1 | >16 | >16 |
| *S. aureus* (MRSA) | 5666 | 1 | 0.5 | 1 | >16 | >16 |
| *S. aureus* (MRSA) | 5702 | 1 | 0.5 | 1 | 16 | >16 |
| *S. aureus* (MRSA) | 5999 | 1 | 0.5 | 1 | 0.5 | >16 |
| *S. aureus* (MRSA) | 6165 | 1 | 1 | 1 | >16 | >16 |
| *S. aureus* (MRSA) | 6169 | 1 | 0.5 | 0.5 | 16 | >16 |
| *S. aureus* (MRSA) | 6174 | 1 | 0.5 | 1 | 8 | >16 |
| *S. aureus* (MRSA) | 6311 | 1 | 1 | 1 | >16 | >16 |
| *S. aureus* (MRSA) | 6312 | 2 | 0.5 | 1 | 16 | >16 |
| *S. aureus* (MRSA) | 6313 | 1 | 0.5 | 1 | >16 | >16 |
| *S. aureus* (MRSA) | 6315 | 1 | 1 | 1 | >16 | >16 |
| *S. aureus* (MRSA) | 6320 | 1 | 1 | 1 | >16 | >16 |
| *S. aureus* (MRSA) | 6498 | 2 | 0.5 | 1 | >16 | 0.5 |
| *S. aureus* (MRSA) | 6501 | 2 | 0.5 | 1 | >16 | >16 |
| *S. aureus* (MRSA) | 6505 | 1 | 0.5 | 1 | >16 | >16 |
| *S. aureus* (MRSA) | 6506 | 2 | 0.5 | 1 | 0.5 | 0.5 |
| *S. aureus* (MSSA) | 2127 | 1 | 1 | >16 | 0.25 | 0.12 |
| *S. aureus* (MSSA) | 6167 | 2 | 0.5 | 1 | 0.25 | >16 |
| *S. aureus* (MSSA) | 6168 | 1 | 0.5 | 2 | 1 | 0.25 |
| *S. aureus* (MSSA) | 6170 | 1 | 0.5 | 2 | 1 | 0.5 |
| *S. aureus* (MSSA) | 6316 | 2 | 1 | 2 | >16 | >16 |
| *S. aureus* (MSSA) | 6317 | 1 | 0.5 | 1 | 0.5 | 0.5 |
| *S. aureus* (MSSA) | 6318 | 1 | 0.5 | 2 | 0.5 | >16 |
| *S. aureus* (MSSA) | 6499 | 2 | 0.5 | 1 | 0.5 | >16 |
| *S. aureus* (MSSA) | 6500 | 1 | 0.5 | 1 | 0.25 | 8 |
| *S. aureus* (MSSA) | 6502 | 1 | 0.5 | 1 | 1 | 0.25 |
| *S. aureus* (VISA) | NRS1/1723 | 2 | 8 | 0.25 | >16 | >16 |
| *S. aureus* (VISA) | 1724 | 2 | 4 | 0.25 | 1 | 0.25 |
| *S. aureus* (VISA) | 2012 | 2 | 8 | 1 | >16 | >16 |
| *S. aureus* (VISA) | 2013 | 2 | 4 | 0.25 | >16 | >16 |
| *S. aureus* (VISA) | 2014 | 2 | 4 | 0.5 | >16 | >16 |
| *S. aureus* (VISA) | 2015 | 2 | 4 | 0.06 | >16 | >16 |
| *S. aureus* (VISA) | 2016 | 2 | 4 | 0.25 | >16 | >16 |
| *S. aureus* (VISA) | 2017 | 2 | 8 | 0.5 | 0.5 | 0.25 |
| *S. aureus* (VISA) | 2018 | 2 | 4 | 0.12 | >16 | >16 |
| *S. aureus* (VISA) | 2019 | 1 | 4 | 4 | >16 | >16 |
| *S. aureus* (VISA) | 2123 | 2 | 8 | 0.25 | >16 | >16 |

## Supplementary Table 2 (continued)

| Organism (Phenotype) | Culture Number/Strain | DNM0547 | vancomycin | mupirocin | ciprofloxacin | erythromycin |
|---|---|---|---|---|---|---|
| *S. aureus* (VISA) | 2124 | 2 | 4 | 0.12 | >16 | >16 |
| *S. aureus* (VISA) | 4658 | 2 | 4 | 0.5 | >16 | >16 |
| *S. aureus* (VISA) | 4660 | 2 | 4 | 0.25 | 0.5 | 0.5 |
| *S. aureus* (VISA) | 4661 | 2 | 8 | 0.25 | >16 | 8 |
| *S. aureus* (VISA) | 4662 | 2 | 4 | 16 | >16 | >16 |
| *S. aureus* (VISA) | 4664 | 2 | 4 | 0.25 | >16 | >16 |
| *S. aureus* (VISA) | 4665 | 2 | 4 | 16 | >16 | >16 |
| *S. aureus* (VISA) | 4666 | 2 | 4 | >16 | >16 | >16 |
| *S. aureus* (VISA) | 4667 | 1 | 4 | 0.25 | >16 | >16 |
| *S. aureus* (VISA) | 4668 | 1 | 4 | 0.25 | >16 | >16 |
| *S. aureus* (VISA) | 4672 | 1 | 2 | 0.06 | 4 | 4 |
| *S. aureus* (VISA) | NRS2/5989 | 2 | 1 | 0.25 | >16 | >16 |
| *S. epidermidis* (VISE) | 2020 | 8 | 4 | 0.5 | 0.25 | >16 |
| *S. epidermidis* (VISE) | 2021 | 1 | 8 | >16 | 8 | >16 |
| *S. epidermidis* (VISE) | 2022 | 2 | 8 | >16 | 0.25 | >16 |
| *S. epidermidis* (VISE) | 2023 | 4 | 8 | >16 | >16 | >16 |
| *S. epidermidis* (VISE) | 2024 | 32 | 2 | 0.5 | >16 | >16 |
| *S. epidermidis* (VISE) | 2025 | 4 | 8 | >16 | >16 | >16 |
| *S. epidermidis* (MSSE) | 3234 | 4 | 2 | 0.25 | 0.25 | 0.5 |
| *S. epidermidis* (MSSE) | 3602 | 2 | 1 | 0.25 | 0.25 | 0.25 |
| *S. epidermidis* (MSSE) | 3609 | 4 | 1 | 0.25 | 0.25 | 0.25 |
| *S. epidermidis* (MSSE) | 3617 | 1 | 2 | 0.25 | 0.12 | 0.25 |
| *S. epidermidis* (MSSE) | 3620 | 2 | 2 | 0.25 | 0.12 | 0.25 |
| *S. epidermidis* (MSSE) | 3621 | 1 | 2 | 0.25 | 0.25 | 0.5 |
| *S. epidermidis* (MRSE) | 3679 | 2 | 2 | 0.5 | >16 | >16 |
| *S. epidermidis* (MRSE) | 3680 | 2 | 1 | >16 | >16 | >16 |
| *S. epidermidis* (MRSE) | 3681 | 1 | 2 | >16 | 8 | >16 |
| *S. epidermidis* (MRSE) | 3683 | 2 | 4 | >16 | 0.25 | >16 |
| *S. epidermidis* (MRSE) | 3745 | 8 | 1 | 0.25 | 4 | >16 |
| *S. epidermidis* (MRSE) | 3746 | 2 | 2 | >16 | >16 | >16 |
| *S. epidermidis* (MRSE) | 3747 | 2 | 2 | >16 | 0.12 | >16 |
| *E. faecalis* (VSE) | 1170 | 2 | 0.5 | >16 | 1 | 0.25 |
| *E. faecalis* (VSE) | 3899 | 1 | 1 | >16 | >16 | 2 |
| *E. faecalis* (VSE) | 4909 | 1 | 0.5 | 1 | >16 | 2 |
| *E. faecalis* (VSE) | 4044 | 2 | 0.5 | 2 | 1 | 4 |
| *E. faecalis* (VSE) | 4182 | 2 | 0.5 | 1 | 1 | 4 |
| *E. faecalis* (VSE) | 3194 | 2 | 1 | >16 | 1 | >16 |
| *E. faecalis* (VSE) | 3901 | 2 | 1 | >16 | 1 | >16 |
| *E. faecalis* (VSE) | 4181 | 1 | 1 | 1 | 2 | >16 |
| *E. faecalis* (VSE) | 413 | 2 | 1 | >16 | >16 | >16 |
| *E. faecalis* (VSE) | 3840 | 2 | 0.5 | 0.5 | >16 | >16 |
| *E. faecalis* (VRE) | 3849 | 2 | >64 | 0.5 | >16 | >16 |
| *E. faecalis* (VRE) | 4026 | 2 | >64 | 1 | >16 | >16 |
| *E. faecalis* (VRE) | 3846 | 2 | >64 | 1 | >16 | >16 |
| *E. faecalis* (VRE) | 4047 | 2 | >64 | 1 | >16 | >16 |
| *E. faecalis* (VRE) | 6743 | 2 | >64 | 1 | >16 | >16 |
| *E. faecalis* (VRE) | 202 | 2 | 8 | >16 | 0.5 | >16 |
| *E. faecalis* (VRE) | 4212 | 2 | 32 | >16 | >16 | >16 |
| *E. faecalis* (VRE) | 4158 | 1 | >64 | >16 | >16 | >16 |
| *E. faecalis* (VRE) | 3835 | 1 | >64 | >16 | >16 | >16 |
| *E. faecalis* (VRE) | 3826 | 2 | >64 | >16 | >16 | >16 |

**Supplementary Table 3**. **Minimum inhibitory concentration (MIC; μg/ml) of DNM compounds that show potential synergy in combination with colistin**. For drug combination experiments the DNM compounds were used at 5 μg and 0.5 μg and the MIC of colistin was determined.

| Compound | *K. pneumoniae* | *A. baumannii* | *E. coli* | *P. aeruginosa* |
|---|---|---|---|---|
| | **MMX8390** | **ATCC 19606** | **ATCC 25922** | **ATCC 27853** |
| | | | | |
| colistin | 16 | 0.25 | 0.25 (0.25-2) | 0.25 (0.25-4) |
| DNM0650 | >64 | >64 | >64 | >64 |
| DNM0652 | >64 | >64 | >64 | >64 |
| DNM0658 | >64 | >64 | >64 | >64 |
| DNM0755 | >64 | >64 | >64 | >64 |
| colistin/DNM650 - 5 ug/ml | 1 | < 0.06 | < 0.06 | 0.25 |
| colistin/DNM650 - 0.5 ug/ml | 2 | 0.25 | < 0.06 | 0.25 |
| colistin/DNM652 - 5 ug/ml | 0.25 | < 0.06 | < 0.06 | 0.25 |
| colistin/DNM652 - 0.5 ug/ml | 2 | < 0.06 | < 0.06 | 0.25 |
| colistin/DNM658 - 5 ug/ml | 0.5 | < 0.06 | < 0.06 | 0.25 |
| colistin/DNM658 - 0.5 ug/ml | 2 | < 0.06 | < 0.06 | 0.25 |
| colistin/DNM755 - 5 ug/ml | 0.5 | < 0.06 | < 0.06 | 0.12 |
| colistin/DNM755 - 0.5 ug/ml | 16 | < 0.06 | 0.12 | 0.25 |

**Supplementary Table 4**. **Synergy of DNM0652 in combination with colistin against *K. pneumoniae*.** Fractional inhibitory concentration index (FICI) values were determined for DNM0652 in combination with colistin. Synergy was observed against each *K. pneumoniae* strain.

| Strain | Mean FICI |
|---|---|
| *K. pneumoniae* (FDA-CDC AR-BANK0040) | 0.41 |
| *K. pneumoniae* (FDA-CDC AR-BANK0046) | 0.4 |
| *K. pneumoniae* (FDA-CDC AR-BANK0047) | 0.3 |
| *K. pneumoniae* (FDA-CDC AR-BANK0087) | 0.38 |
| *K. pneumoniae* (FDA-CDC AR-BANK0097) | 0.34 |
| *K. pneumoniae* (FDA-CDC AR-BANK0106) | 0.32 |
| *K. pneumoniae* (FDA-CDC AR-BANK0109) | 0.35 |
| *K. pneumoniae* (FDA-CDC AR-BANK0125) | 0.32 |
| *K. pneumoniae* (FDA-CDC AR-BANK0364) | 0.29 |
| *K. pneumoniae* (FDA-CDC AR-BANK0454) | 0.28 |
| *K. pneumoniae* (ATCC 700603) | 0.51 |
| *K. pneumoniae* (ATCC 13883) | 0.19 |
